# International practice patterns and factors associated with non-conventional hemodialysis utilization

**DOI:** 10.1186/1471-2369-12-66

**Published:** 2011-12-05

**Authors:** Nathan Allen, Daniel Schwartz, Paul Komenda, Robert P Pauly, Deborah Zimmerman, Gemini Tanna, Jeffery Schiff, Claudio Rigatto, Manish M Sood

**Affiliations:** 1 Department of Medicine, Section of Nephrology, St Boniface Hospital, University of Manitoba, 409 Tache Avenue, Winnipeg, R2H 2A6, Canada; 2 Department of Medicine, Section of Nephrology, Abbotsford Regional Hospital, University of British Columbia, 32900 Marshall Road, Abbotsford, V3T 5H5, Canada; 3 Department of Medicine, Section of Nephrology, University of Alberta, 112th Street, Edmonton, T6G 2G3, Canada; 4 Department of Medicine, Section of Nephrology, The Ottawa Hospital, University of Ottawa. 1967 Riverside Drive, Ottawa, K1H 7W9, Canada; 5 Department of Medicine, Section of Nephrology, Sunnybrook Hospital, University of Toronto, 2075 Bayview Avenue, Toronto, M4N 3M5, Canada; 6 Department of Medicine, Section of Nephrology, University Health Network, University of Toronto, 200 Elizabeth Street, Toronto, M5G 2C4, Canada; 7 Department of Medicine, Section of Nephrology, Seven Oaks Hospital, University of Manitoba, 2300 McPhillips Street, Winnipeg, R2V 3M3, Canada

## Abstract

**Background:**

The purpose of our study was to determine characteristics that influence the utilization of non-conventional hemodialysis (NCHD) therapies and its subtypes (nocturnal (NHD), short daily (SDHD), long conventional (LCHD) and conventional hemodialysis (CHD) as well as provider attitudes regarding the evidence for NCHD use.

**Methods:**

An international cohort of subscribers of a nephrology education website http://www.nephrologynow.com was invited to participate in an online survey. Non-conventional hemodialysis was defined as any forms of hemodialysis delivered > 3 treatments per week and/or > 4 hours per session. NHD and SDHD included both home and in-centre. Respondents were categorized as CHD if their centre only offered conventional thrice weekly hemodialysis. Variables associated with NCHD and its subtypes were determined using multivariate logistic regression analysis. The survey assessed multiple domains regarding NCHD including reasons for initiating and discontinuing, for not offering and attitudes regarding evidence.

**Results:**

544 surveys were completed leading to a 15.6% response rate. The final cohort was limited to 311 physicians. Dialysis modalities utilized among the respondents were as follows: NCHD194 (62.4%), NHD 83 (26.7%), SDHD 107 (34.4%), LCHD 81 (26%) and CHD 117 (37.6%). The geographic regions of participants were as follows: 11.9% Canada, 26.7% USA, 21.5% Europe, 6.1% Australia/New Zealand, 10% Africa/Middle East, 10.9% Asia and 12.9% South America. Variables associated with NCHD utilization included NCHD training (OR 2.47 CI 1.25-4.16), government physician reimbursement (OR 2.66, CI 1.11-6.40), practicing at an academic centre (OR 2.28 CI 1.25-4.16), higher national health care expenditure and number of ESRD patients per centre. Hemodialysis providers with patients on NCHD were significantly more likely to agree with the statements that NCHD improves quality of life, improves nutritional status, reduces EPO requirements and is cost effective. The most common reasons to initiate NCHD were driven by patient preference and the desire to improve volume control and global health outcomes.

**Conclusion:**

Physician attitudes toward the evidence for NCHD differ significantly between NCHD providers and conventional HD providers. Interventions and health policy targeting these areas along with increased physician education and training in NCHD modalities may be effective in increasing its utilization.

## Background

The emergence of non-conventional hemodialysis therapies (NCHD), such as nocturnal HD (NHD), short daily HD (SDHD) and long, thrice weekly (LHD), as dialysis modalities have resulted in changes to the dialysis prescription on an individual basis. Cardiovascular benefits, achievement of phosphate and neutral fluid balance and improvements in quality of life have resulted in a paradigm shift supporting the increased usage and promotion of NCHD [1,2]. From a health economics perspective, subtypes of NCHD such as nocturnal HD have been shown to be more cost effective than conventional in-centre HD [3-5]. Despite all these benefits, NCHD continues to have variable uptake and utilization in many countries.

The decision to initiate non-conventional hemodialysis is based on several complex patient, physician institutional, and health care payer factors. Barriers to NCHD at the level of the patient and health care delivery system have been explored in a few regional studies and mostly among high income nations [6-8]. Physician-specific reasons for not utilizing this modality have not been well defined, despite the fact that physicians themselves estimate that approximately 20% of the renal replacement population would be suitable non-conventional or nocturnal HD [9]. This is especially important as in many centres, the decision to initiate non-conventional HD is most commonly physician driven.

The aim of the present study is to evaluate physician and practice characteristics that influence the utilization of non-conventional HD and its subtypes of nocturnal, short daily and long conventional HD in an international cohort and characterize attitudes regarding initiation of and the evidence for NCHD use.

## Methods

Our survey was distributed to Nephrology Now subscribers with survey methodology and population surveyed described elsewhere (please see additional file 1 for the original survey instrument) [10,11]. In brief, Nephrology Now is a non-profit, online, free email alert service that selects nephrology journal articles and incorporates them into a monthly mailing list http://www.nephrologynow.com. The monthly mailing list includes the 15-30 articles with title, authors, journal and date of publication, a brief summary and a link to the article abstract. As of April 1, 2011, Nephrology Now has 3, 498 subscribers from over 150 countries.

### Study Population

All subscribers to the Nephrology now monthly mailing list were contacted by email in the winter of 2011 and invited to participate in a survey. A total of 3 reminder emails were sent to all subscribers inviting them to participate. Consent was based on participation in the survey. University of Manitoba regional ethics board approved this study.

### Survey Design

The survey was developed, implemented and tracked using Survey monkey http://www.surveymonkey.com. Survey monkey reports the number of survey emails that were opened (click rate). MMS, DS, PK, NA were primarily involved in the question development and survey design. Pre-testing was completed by the survey designers and other members of the Nephrology Now editorial board.

### Outcome and Assessment of Physician Attitudes

The main outcomes measured were utilization of non-conventional hemodialysis which was defined as "any forms of hemodialysis delivered > 3 treatments per week and/or > 4 hours per session" and its subtypes, nocturnal, short daily and long conventional hemodialysis. NHD and SDHD included both home and in-centre and each respondent could identify more than one dialysis modality available at their centre except for conventional hemodialysis (CHD) which included respondents who offered only CHD at their centre. Physician attitudes towards efficacy/evidence, indications for initiating, perceived barriers to and methods to improve education about NCHD were assessed and summarized as proportions.

### Data Analysis

Demographic, practice and health system differences among providers and non-providers of NCHD and NHD were determined by student's t-test for continuous variables, chi-square for dichotomous variables and the Mann-Whitney U test for non-parametric variables. Only respondents who were physicians were included in the final analysis. P values < 0.05 were considered significant. All analyses were conducted using PASW v18 (Texas, USA).

**Figure 1:**
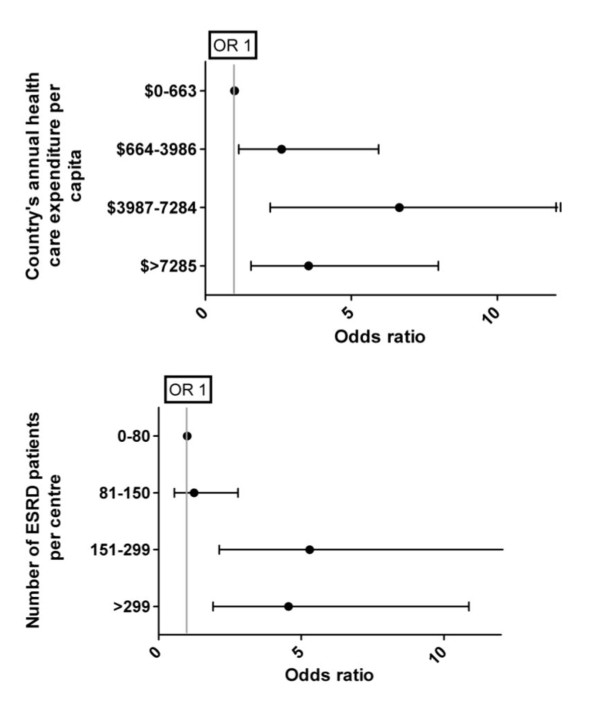
**Relationship between Non-conventional hemodialysis and a) National health care expenditure and b) Number of ESRD patients per centre**. Adjusted for academic centre, training, type of reimbursement, country's health care expenditure.

Covariates associated with the outcomes of NCHD and its subtypes were assessed for association by logistic regression. A multivariate model was created with covariate inclusion based in statistical significance on univariate analysis or an *a priori *belief of their clinical relevance. Statistically significant changes in the -2 log likelihood were used to determine variables retained in the final model. Number of ESRD patients, total health expenditure per capita and percentage of gross domestic product spent on health care per capita analyzed as continuous variables though divided into quartiles for illustrative purposes (See Figure 1a and 1b).

National data on the total health expenditure per capita and percentage of gross domestic product spent on health care per capita was obtained from 2008 core health indicators report at the World Health Organization [12]. The report included health statistics for the 193 WHO member states.

## Results

A total of 3498 subscribers were invited to participate of which 1329 surveys were opened (37.9% mean click rate) and 544 were completed leading to a 15.6% response rate. The 544 respondents consisted of physicians and non-physicians and the final cohort included in the analysis was limited to 311 physicians. Of note, no physician respondents were excluded, only non-physicians. Dialysis modalities utilized among the respondents were as follows: NCHD 194 (62.4%), NHD 83 (26.7%), SDHD 107 (34.4%), LCHD 81 (26%) and CHD 117 (37.6%).

Physician and practice characteristics are summarized in table 1 and 2. The majorities of physicians were attending staff (83.8%) and worked in urban centres (91.3%). Slightly over half worked in academic centres (58.8%) and regularly used PDAs (57.6%). All lengths of practice and geographic regions were represented with the majority of respondents from Europe and USA (21.5% and 26.7% respectively) and few from Australia and New Zealand (6.1%). The majority of physicians worked in centres with catchment populations of 500, 000 to 5 million with fewer physicians working in centres with smaller (8.4%) and larger (4.7%) catchment areas. Regarding NCHD reimbursement, 74 (23.8%) physicians were primarily reimbursed by the patient, 102 (32.8%) by private insurance, and 266 (85.5%) by the government or public insurance. Greater than one form of reimbursement was possible per physician.

**Table 1:** Characteristics of the study cohort stratified by non-conventional hemodialysis (NCHD) and conventional hemodialysis (CHD) only presented as proportions with actual counts in parentheses.

Characteristic	Total %(N)	NCHD %(N)	CHD only %(N)	P
**Total**		62.4(194)	37.6(117)	

**Level of Training**				

Staff Physician	83.8 (259)	85.1(165)	81.7(94)	0.5

Fellow	9.6 (30)	7.7(15)	13(15)	0.2

Resident	6.5 (20)	7.2(14)	5.2(6)	0.6

**Geographic Region**				

Canada	11.9 (37)	16.5(32)	4.3(5)	0.001

USA	26.7(83)	32(62)	17.9(21)	0.008

Europe	21.5 (67)	22.7(44)	19.7(23)	0.6

Australia/New Zealand	6.1 (19)	8.8(17)	1.7(2)	0.01

Africa/Middle East	10 (31)	8.2(16)	12.8(15)	0.2

South America	12.9 (40)	7.2(14)	22.2(26)	< 0.001

Asia	10.9 (34)	4.6(9)	21.4(25)	< 0.001

**Smartphone use/personal digital assistant (PDA)**	57.6 (179)	61.9(120)	50.4(33)	0.06

**Academic Centre**	58.8 (181)	66.1(127)	46.6(54)	0.001

**Urban**	91.3 (284)	92.3(179)	89.7(105)	0.5

**Catchment population**				

< 100 k	8.4 (26)	6.2(12)	12(14)	0.09

100-500 k	37.0 (115)	35.6(69)	39.3(46)	0.5

500-1 million	28.3 (88)	32.5(63)	21.4(25)	0.04

1-5 million	21.5 (67)	21.1(41)	22.2(26)	0.9

> 5 million	4.7 (18)	4.6(9)	5.1(6)	1.0

**Length of Practice**				

1-5 years	29.3 (91)	29.4(57)	29.1(34)	1.0

6-10 years	22.2(69)	25.3(49)	17.1(20)	0.1

11-15 years	13.5(42)	13.4(26)	13.7(16)	1.0

16-20	9.6(30)	13.7(16)	13.4(26)	0.8

> 20	25.4 (79)	22.7(44)	29.9(35)	0.1

**Frequent dialysis Reimbursement**				

Patient primary payer	23.8(74)	23.3(45)	24.8(29)	0.8

Government	85.5(266)	90.7(176)	76.8(90)	0.001

Private insurance	32.8(102)	34.5(67)	29.9(35)	0.5

**National healthcare expenditure per capita (median (IQR) in US dollars**	3986 (6703)	4409(3418)	663(3619)	< 0.001

**National healthcare expenditure as percentage GDP (median (IQR))**	8.9(9)	10.1(7.3)	8(5.5)	< 0.001

**Number of ESRD pts. (median)**	150(220)	200(240)	108(138)	< 0.001

**Table 2:** Characteristics of the study cohort stratified by nocturnal (NHD), short daily (SDHD), long conventional (LCHD) and conventional hemodialysis (CHD) prescribers presented as proportions with actual counts in parentheses.

Characteristic	NHD %(N)	SDHD %(N)	LCHD %(N)	CHD only %(N)
**Total**	26.7(83)	34.4(107)	26(81)	37.6(117)

**Level of Training**				

Staff Physician	85.5(71)	84.1(90)	81.5(66)	81.7(94)

Fellow	10.8(9)	8.4(9)	12.3(10)	13(15)

Resident	3.6(3)	7.5(8)	6.2(5)	5.2(6)

**Geographic Region**				

Canada	27.7(23)	19.6(21)	6.2(5)	4.3(5)

USA	31.3(26)	35.5(38)	23.5(19)	17.9(21)

Europe	16.9(14)	23.4(25)	29.6(24)	19.7(23)

Australia/New Zealand	14.5(12)	7.5(8)	8.6(7)	1.7(2)

Africa/Middle East	3.6(3)	4.7(5)	12.3(10)	12.8(15)

South America	2.4(2)	6.5(7)	11.1(9)	22.2(26)

Asia	3.6(3)	2.8(3)	8.6(7)	21.4(25)

**Smartphone use/personal digital assistant (PDA)**	63.9(53)	61.7(66)	58(47)	50.4(33)

**Academic Centre**	64.6(53)	68.9(73)	67.5(54)	46.6(54)

**Urban**	94(78)	94.4(101)	97.5(79)	89.7(105)

**Catchment population**				

< 100 k	2.4(2)	0(0)	2.5(2)	12(14)

100-500 k	39.8(33)	39.3(42)	37(30)	39.3(46)

500-1 million	32.5(27)	34.6(37)	28.4(23)	21.4(25)

1-5 million	22.9(19)	22.4(24)	29.6(24)	22.2(26)

> 5 million	2.4(2)	3.7(4)	2.5(2)	5.1(6)

**Length of Practice**				

1-5 years	24.1(20)	32.7(35)	21(17)	29.1(34)

6-10 years	27.7(23)	25.2(27)	23.5(19)	17.1(20)

11-15 years	21.7(18)	11.2(12)	16(13)	13.7(16)

16-20	8.4(7)	7.5(8)	13.6(11)	13.4(26)

> 20	18.1(15)	23.4(25)	25.9(21)	29.9(35)

**Frequent dialysis Reimbursement**				

Patient primary payer	21.7(18)	19.6(21)	24.7(20)	24.8(29)

Government	91.6(76)	88.8(95)	82.7(67)	76.8(90)

Private insurance	38.6(32)	35.5(38)	34.6(28)	29.9(35)

**National healthcare expenditure per capita (median (IQR) in US dollars)**	4409 (3299)	4409(3418)	3867(6262)	663(3619)

**National healthcare expenditure as percentage GDP (median (IQR))**	10.1(6.8)	10.1(7.3)	8.9(8.1)	8(5.5)

**Number of ESRD pts. (median (IQR))**	242(275)	240(250)	200(254)	108(138)

Physicians practicing in Canada, Australia/New Zealand, in academic centres, with increasing number of ESRD patients per centre and in catchment areas of 500, 000 to 1 million had significantly more patients on NCHD. Countries with high national health care expenditures, high percentage of GDP expenditure on health care and government or public physicians' reimbursement were more likely to utilize NCHD.

In contrast, CHD only was more common in Asia, South America and Africa/Middle East and relatively rare in Canada and Australia/New Zealand. Furthermore, centres with CHD only were less likely to be academic centres, be publically reimbursed, and had less ESRD patients total and lower health care expenditure. LCHD was more common USA and Europe as opposed to Canada

Characteristics associated with NCHD and its subtypes are presented in tables 3 and 4. In multivariate modeling, practicing in an academic centre, NCHD training, public or government physician reimbursement, increasing national health care expenditures and number of patients with ESRD per centre were independently associated with NCHD utilization. Among the subtypes of dialysis, the number of ESRD patients per centre was consistently associated with all subtypes of HD. NHD and SDHD were also associated with health care expenditure. The relationship between having patients on NCHD and the number of patients with ESRD per centre and increasing countries health care expenditures are depicted in Figures 1a and 1b.

**Table 3:** Characteristics associated with non-conventional hemodialysis (NCHD) usage in a multivariate logistic regression model.

Characteristic	Odds Ratio	95% CI	P
**Government reimbursement**	2.66	1.11-6.40	0.03

**Patient reimbursement**	1.29	0.61-2.71	0.5

**Private insurance reimbursement**	1.00	0.47-2.09	1.0

**NCHD training**	2.47	1.37-4, 43	0.003

**Practice at an academic centre**	2.28	1.25-4.16	0.007

**Number of ESRD patients at centre (per 10 patient increase)**	1.03	1.01-1.04	0.002

**National health care expenditure**	1.0	1.0-1.0	< 0.001

Health expenditure is in US dollars

**Table 4:** Characteristics associated with nocturnal, short-daily and long conventional hemodialysis usage by multivariate logistic regression.

Characteristic	Odds Ratio	95% CI	P
**Nocturnal hemodialysis:**			

Number of ESRD patients at centre (per 10 patient increase)	1.03	1.01-1.04	< 0.001

National health care expenditure	1.00	1.00-1.00	< 0.001

**Short daily hemodialysis:**			

Practice at an academic centre	1.77	0.97-3.22	0.06

Number of ESRD patients at centre (per 10 patient increase)	1.02	1.01-1.04	< 0.001

National health care expenditure	1.0	1.0-1.0	< 0.001

**Long conventional hemodialysis:**			

Number of ESRD patients at centre (per 10 patient increase)	1.01	1.0-1.02	0.08

Health expenditure is in US dollars

**Figure 2:**
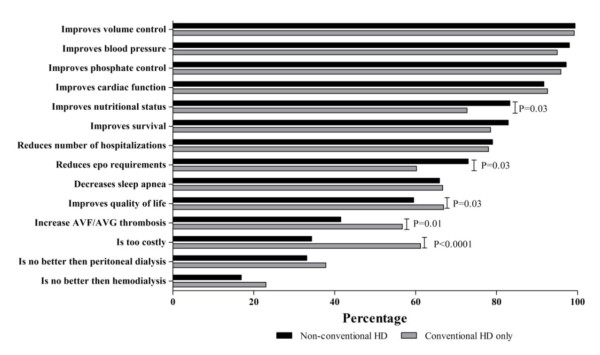
**Physicians attitudes towards the evidence for non-conventional hemodialysis stratified by use or non-use**.

**Figure 3:**
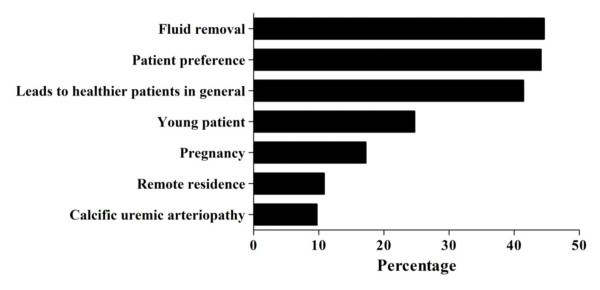
**Commonly cited indications for initiation of non-conventional hemodialysis among physicians**.

The results of physician attitudes towards the efficacy and evidence for NCHD are summarized in Figures 2 and 3. There was near universal agreement that NCHD improves phosphate control, improves BP control and improves volume status with greater than 95% of physicians agreeing with each of these statements. In addition, the majority of physicians agreed that NCHD improves survival (81%), reduces hospitalizations (79%), and improves sleep apnea (66%) whether or not they currently had patients on NCHD. Physicians with patients on NCHD were significantly more likely to agree with the statements that it improves quality of life, improves nutritional status, and reduces EPO requirements. They were significantly less likely to agree with the statements that NCHD increases fistula/graft thrombosis and is too costly.

Among providers of NCHD, the most common reasons for HD providers (see Figure 3) were to improve volume control (44.6%), patient preference (44.1%) and the belief it improves health in general (41.4%). Other reasons cited include younger patients (24.7%), pregnancy (17.2%), calcific uremic arteriopathy (calciphylaxis) (9.7%) or remote patient residence (10.8%).

**Figure 4:**
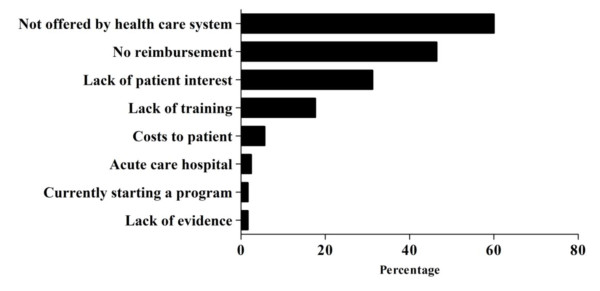
**Commonly cited barriers to non-conventional hemodialysis usage among users and non-users**.

The reasons for not offering NCHD are summarized in Figure 4. Lack of availability (60.0%), lack of reimbursement (46.4%), lack of patient interest (31.2%) and lack of adequate training (17.6%) were commonly cited. When providers who cited inadequate training as a barrier to offering NCHD were asked for their preferred methods of FHD education online CME (53.9%), conference (56.5%) or local (47.8%) presentation by an expert and a review of the primary literature or journal club (507%) were common responses. Additional fellowship training (25.4%) and smartphone based CME (18.8%) was substantially less desirable.

## Discussion

Non-conventional forms of hemodialysis are underutilized dialysis modalities despite growing evidence of the benefits in improving patient outcomes [1-5,13,14]. Understanding physician and practice characteristics that favorably influence the utilization of NCHD are essential if it is to be more widely adopted. The present study is the first international survey to assess physician and practice specific characteristics associated with NCHD usage. Practicing in an academic centre, NCHD training, public or government physician reimbursement increases in national health care expenditures and numbers of patients with ESRD per centre were all independently associated with NCHD utilization.

The association of national health care on increasing NCHD utilization in our cohort is in contrast to recent findings by MacGregor *et al *who found no relationship between expenditure and NCHD in a registry based study [9]. Several factors could explain this discrepancy. The present study examined NCHD and all of its subtypes including NHD and SDHD. Increases in national health care expenditure were associated with NHD and SDHD specifically but not LCHD. In addition, the present study included nephrologists from Africa, Asia and South America while the MacGregor study included only data from high and middle income countries in North America, Europe and Australia/New Zealand [9]. Third the methodology in the two studies differed significantly and may have led to disparate results; the present study was designed to evaluate physician and practice patterns while the prior study was population based.

The relationship between health care expenditures and NCHD was not linear. As shown in Figure 1a, NCHD utilization was significantly lower in countries in the lowest quartile of health care expenditures but there was no significant difference in countries in the top three quartiles. In fact, countries in the highest quartile had a subjective decrease in the utilization of NCHD. Two observations may explain these findings. First, while NCHD may be more cost-effective in the long-term it does require higher up-front costs and requires a significant amount of infrastructure to exist [7]. This infrastructure often does not exist in countries with the lowest per capita health care expenditures and the higher up-front costs are likely prohibitive. Second, the finding of a subjective decrease in the utilization of FHD in countries in the highest quartile is likely explained by an over-representation of nephrologists from the USA in the present study. Despite the high cost of healthcare in the USA, the rates of NCHD remain quite low primarily due to lack of reimbursement schedules that facilitate home based, or more frequent therapies [15]. In addition, other wealthy countries with higher rates of utilization of NCHD such as Australia and New Zealand were under-represented in the present study. Nevertheless there appears to be a necessary minimum national health care expenditure required to allow for NCHD after which other factors such as reimbursement and NCHD training appear to influence NCHD usage.

The finding of centre size being associated with more utilization of NCHD while novel is not surprising and also did not appear to be linear. There was significantly more NCHD in centres with greater than 150 ESRD patients than in those with less. Once this threshold was reached there was no further increase in NCHD utilization with increased ESRD patient numbers. Similar findings have been demonstrated in peritoneal dialysis usage where smaller centres had lower adherence to guidelines, particularly in the area of continuous quality initiatives [10]. Similar to continuous quality initiatives which become more difficult and less economically feasible in a centre with a small number of patients on peritoneal dialysis, NCHD requires infrastructure and upfront costs and patient training may not be possible in centres with small amounts of ESRD patients. In addition, approximately 6-8% of patients exit home hemodialysis programs per year [7]. Losing patients early on in this modality significantly increases per patient cost and this added cost would be more difficult to absorb in smaller centres with fewer patients. It would appear that both economies of scale and economies of scope are more easily attained with larger programs delivering ESRD care.

NCHD usage is influenced by physician remuneration. The lack of remuneration for NCHD was commonly cited as a barrier and was second only to NCHD not being offered by the health care system. Public healthcare systems in Australia, New Zealand, the Netherlands and Canada have instituted programs that encourage and incentivize NCHD [8]. In Australia, an additional payment of 128$ per month is provided to physicians managing patients with home dialysis modalities [9]. Based on the results of the present study, physician remuneration and incentives appear to increase utilization of NCHD.

NCHD was associated with practicing in academic centres and this is likely related to the impression by some that NCHD was considered an experimental modality; most patients on FNCHD were managed out of large research institutions and/or were part of studies on the potential benefits and or feasibility of NCHD. As evidence demonstrating a significant clinical benefit for NCHD mounts and this therapy is adopted by different healthcare systems these differences are likely to become less pronounced.

Physician training in NCHD impacted utilization in our study. Until recently, it is unlikely that physicians practicing outside a few individual institutions, such as Tassin, France would have had any exposure to NCHD [16]. In fact, it was not until approximately 15 years ago and the pioneering work by Uldall and colleagues in Toronto, Canada that there was resurgence in interest in NCHD [17]. Few physicians have formal training in the safe prescription and patient selection paradigms for NCHD, limiting physician comfort and exposure to the potential benefits of this modality. Funding and active knowledge dissemination regarding NCHD is imperative if any healthcare system wishes to increase the amount of patients on NCHD. According to the present study the preferred methods of achieving this goal would be through online CME, presentations by experts in NCHD and through journal club activities.

Finally, the attitudes and opinions of physicians towards the evidence for NCHD are encouraging for the future of this modality. There was overall acceptance among physicians regarding the clinical advantages of NCHD. There was nearly universal agreement that NCHD improves phosphate control, BP control and volume status; outcomes evaluated in 2 recent RCTs [1,2]. There was significant disagreement about the evidence in certain areas. In particular, there was disagreement in the areas of quality of life, nutritional status, erythropoietin requirements and graft thrombosis indicating the need for further research in these areas. At present evidence remains equivocal, specifically in the area of complications related to vascular access [1,2]. It should be noted that Physicians may be reluctant to prescribe NCHD with concerns over the increase in interventions and use of vascular access. Increased usage of vascular access in turn may lead to associated complications and incur health care expenditures. An RCT powered to demonstrate a mortality difference in NCHD versus conventional HD is unlikely to ever be performed. The recently completed NIH funded Frequent Hemodialysis Network RCT in this area encountered significant logistical barriers to recruitment using surrogate outcomes. With the majority of surrogate outcomes in the existing observational and randomized trials favoring NCHD, the utility of long, expensive RCT's with mortality as a primary endpoint is questionable given the fact that most of the costing literature in this area have shown home NCHD to be at least cost neutral, if not cost saving.

Our study has certain limitations. First it was a voluntary survey and is therefore prone to selection bias as physicians with no interest in NCHD are less likely to respond. Our survey depended on self-reported data, the reliability of which has been questioned and is prone to recall bias. Our overall response rate was low at 15.6%. This is not uncommon in internet-based surveys and we attempted to improve our survey methodology by identifying how many individuals actually opened the email (click rate). This is important as many email surveys may be discarded to junk mail without the contents of the email and purpose of the survey appropriately conveyed to subscribers. We included relatively crude expenditure metrics such as National healthcare expenditure that may not be sensitive to a country's efficiencies or capture funding directed towards specific, specialized health initiatives. Physician attitudes and opinions were assessed only towards NCHD and not towards its individual subtypes such as NHD or SDHD. This limited our ability to identify factors associated with NHD, SDHD and LCHD. The survey results were limited to physicians and did not include health policy administrators or Allied health, both of whom would likely influence NCHD utilization. Finally, as previously discussed, there may have been over-representation of physicians from the USA and Europe and under-representation from New Zealand and Australia.

## Conclusions

The present study is the first to assess physician and practice characteristics associated with the utilization of NCHD in an international cohort. International variations in the use of NCHD are influenced by training, practicing in an academic centre, centre size, mode of reimbursement and by national health care expenditures. Interventions and health policy targeting these areas as well as increased physician education and training in NCHD modalities may be effective in increasing NCHD utilization. In addition, the present study identifies important areas where significant disagreement exists between practicing physicians in the realm of NCHD. These areas require further exploration if NCHD is to be adopted universally by physicians as a viable dialysis modality.

## Competing interests

The authors declare that they have no competing interests.

## Authors' contributions

NA drafted the manuscript and participated in the study and survey design, DS, PK, RPP, DZ, JS, GT, CR participated in the survey and study design and drafting the manuscript, DS coordinated the online survey development and distribution, MMS participated in drafting the manuscript, study and survey design, study conception and statistical analysis. All authors read and approved of the final manuscript.

## Pre-publication history

The pre-publication history for this paper can be accessed here:

http://www.biomedcentral.com/1471-2369/12/66/prepub

## Supplementary Material

Additional file 1 **An assessment of international non-conventional hemodialysis utilization and practice patterns - Original Survey Instrument**. The original survey questionnaire distributed to subscribers of Nephrology Now. Click here for file
