# Supplementary material for: International practice patterns and factors associated with non-conventional hemodialysis utilization
Source: BMC Nephrol. 2011 Dec 5;12:66. doi: 10.1186/1471-2369-12-66 (PMC3241205; doi:10.1186/1471-2369-12-66)
Supplement: Additional file 1 — An assessment of international non-conventional hemodialysis utilization and practice patterns - Original Survey Instrument. The original survey questionnaire distributed to subscribers of Nephrology Now. [file 1471-2369-12-66-S1.DOC]

**An assessment of international non-conventional hemodialysis utilization and practice patterns.**

**Survey Questions**

**Part 1: Provider characteristics**

1. In what country do you practice?
2. How many years have you been in practice? 0-5 years, 6-10 years, 11-15 years, 16-20 years, >20 years
3. Are you a physician or an allied health care worker? physician. allied health
4. If you are a physician, which would best describe you? Primary Care Physician, Nephrologist, Internal Medicine Specialist other than nephrology, Other
5. If you are a physician at what level of training are you? Resident, Nephrology Fellow, Attending Physician
6. Do you work primarily in an academic centre or in community practice? Academic Centre, Community Practice
7. Is your practice located in an urban (population >100,000) or rural (population <100,000) centre? Urban, Rural
8. What is the catchement area (total population) your institution/hospital provides service to?
9. On average, how many Conferences do you attend annually? 0, 1-2, 3-5, 6-10, >10
10. Do you regularly use a smartphone/PDA device in you practice? yes, no
11. How are you reimbursed? Patient pays, government, other, private insurance, combination government-private, combination government-patient, combination private-patient

**Part 2: Practice characteristics**

1. How many end-stage renal disease patients (both on HD and PD) at your centre?
2. In your centre how many patients are on:
   1. peritoneal dialysis?
   2. conventional HD?
   3. in-centre nocturnal HD?
   4. at home nocturnal HD?
   5. in-centre short daily HD?
   6. at home short daily HD?
   7. long conventional HD (>5 hours three times a week?
   8. a combination of above modalities

3.How many total hours per week (on average) do you prescribe dialysis for the following modalities?

i. peritoneal dialysis?

- 1. conventional HD?
  2. in-centre nocturnal HD?
  3. at home nocturnal HD?
  4. in-centre short daily HD?
  5. at home short daily HD?
  6. long conventional HD?

1. Is there a dedicated home HD program at your centre? yes/no
2. If you have patients on frequent HD:
   1. Did they start on conventional HD or PD prior? yes/no
      1. If yes, why did you switch? Unable to tolerate fluid removal, calciphylaxis, pregnancy, leads to healthier patients, reside remotely, young patient, patient preference, other
   2. Do you use frequent HD as salvage therapy? yes/no
      1. If yes, for what condition? Poor cardiac function, volume removal, hyperphosphatemia, Calcific uremic arteriolopathy, Other
   3. What percentage have following access: catheter, AVG, AVF
   4. Why would you discontinue a pt from frequent HD? Nonadherence, medical indication, social issues, other
3. If you have no patients on NCHD (> 3 times per week), why? No reimbursement, no patient interest, not offered by health care system, lack of physician/allied health training or comfort, other
4. How is NCHD reimbursed at your centre? Private insurance, public insurance, combination of the two, patient pays

**Part 3: Providers attitudes**

Do you agree or disagree with the following statements?

1. NCHD improves patient survival. agree/disagree
2. NCHD improves overall cardiac function. agree/disagree
3. NCHD improves phosphate control. agree/disagree
4. NCHD improves quality of life. agree/disagree
5. NCHD reduces the number of hospitalizations. agree/disagree
6. NCHD improves blood pressure. agree/disagree
7. NCHD reduces erythropoietin receptor agonist requirements. agree/disagree
8. NCHD improves volume control. agree/disagree
9. NCHD improves nutritional status. agree/disagree
10. NCHD reduces the incidence of sleep apnea. agree/disagree
11. NCHD increases fistula/graft thrombosis. agree/disagree
12. NCHD is cost effective. agree/disagree
13. NCHD is too costly. agree/disagree
14. In terms of clinical outcomes, NCHD is no better than in centre HD. agree/disagree
15. In terms of clinical outcomes, NCHD is no better then PD. agree/disagree
